# The Role of Cea and Liver Function Tests in the Detection of Hepatic Metastases From Colo-Rectal Cancer

**DOI:** 10.1155/1990/87874

**Published:** 1990

**Authors:** G. Bonfanti, Luigia Bombelli, F. Bozzetti, R. Doci, L. Gennari, D. Koukouras

**Affiliations:** 1 Istituto Nazionale Tumori V. Venezian Milan 1. 20133 Italy; 2 Santo Andrea Hospital Patrasso University Patrasso Greece; 3 Oncologia Chirurgica ”A” Istituto Nazionale Tumori V. Venezian Milan 1- 20133 Italy

## Abstract

Carcinoembryonic antigen and some liver function tests (alkaline phosphatase, gamma-glutamyl-transpeptidase, lactic dehydrogenase and cholinesterase) were evaluated in patients with primary colorectal cancer in order to define their role in the pre-operative detection of liver metastases.

The records of 278 consecutive patients admitted to the Istituto Nazionale Tumori of Milan between January 1982 and December 1983 who were suffering from primary invasive colo-rectal cancer and who underwent laparotomy were retrospectively analyzed.

At laparotomy, liver metastases were found in 38 pts (13.7%). Considering single tests, CEA was the most sensitive (71%); no single test was found to be reliably predictive, when the result was abnormal. On the contrary, the normal value of each test was associated with a good prediction.

When we considered all the five tests together in the single patient their predictive value, when abnormal, proved to be quite good only if four or five results were abnormal. On the other hand, liver metastases in the presence of all normal tests were found only in two patients, so giving a negative predictive value of about 97%.

So we conclude that, in the lack of an infallable imaging technique for liver evaluation, in the presence of all normal tests any other investigation on the liver could be avoided. However, when liver tests are pathologic, some other imaging technique should be performed in order to supply the surgeon with information about the extent and the spread of the metastases.


*HPB Surgery*, 1990, Vol. 3, pp. 29–37
Reprints available directly from the publisher
Photocopying permitted by license only

© 1990 Harwood Academic Publishers GmbH
Printed in the United Kingdom

### THE ROLE OF CEA AND LIVER FUNCTION TESTS IN THE DETECTION OF HEPATIC METASTASES FROM COLO-RECTAL CANCER

G. BONFANTI, LUIGIA BOMBELLI, F. BOZZETTI, R. DOCI and L. GENNARI

*Istituto Nazionale Tumori, V. Venezian, 1. 20133 Milan - Italy*

D. KOUKOURAS

*Santo Andrea Hospital, Patrasso University, Patrasso, Greece.*

*(Received 20 December 1989)*

Carcinoembryonic antigen and some liver function tests (alkaline phosphatase, gamma-glutamyl-transpeptidase, lactic dehydrogenase and cholinesterase) were evaluated in patients with primary colorectal cancer in order to define their role in the pre-operative detection of liver metastases.

The records of 278 consecutive patients admitted to the Istituto Nazionale Tumori of Milan between January 1982 and December 1983 who were suffering from primary invasive colo-rectal cancer and who underwent laparotomy were retrospectively analyzed.

At laparotomy, liver metastases were found in 38 pts (13.7%). Considering single tests, CEA was the most sensitive (71%); no single test was found to be reliably predictive, when the result was abnormal. On the contrary, the normal value of each test was associated with a good prediction.

When we considered all the five tests together in the single patient their predictive value, when abnormal, proved to be quite good only if four or five results were abnormal. On the other hand, liver metastases in the presence of all normal tests were found only in two patients, so giving a negative predictive value of about 97%.

So we conclude that, in the lack of an infallable imaging technique for liver evaluation, in the presence of all normal tests any other investigation on the liver could be avoided. However, when liver tests are pathologic, some other imaging technique should be performed in order to supply the surgeon with information about the extent and the spread of the metastases.

KEY WORDS: CEA, liver function tests, synchronous liver metastases, colorectal cancer.

### INTRODUCTION

The frequency of liver metastases (LM) at the time of initial surgical exploration for primary colo-rectal cancer is estimated to be between 14% and 25% [1].

The increasing number of favourable reports on the efficacy of a surgical approach [1] and loco-regional chemotherapy [2] for colo-rectal liver metastases increases the importance of the pre-operative detection of secondary lesions.

In spite of the use of various diagnostic modalities such as scintiscan, ultrasonography, CT scan and MNR, the pre-operative demonstration of metastases is often

Address for correspondence: Dr. G. Bonfanti, Oncologia Chirurgica "A", Istituto Nazionale Tumori, V. Venezian 1 - 20133 Milan. Italy.

quite inaccurate, particularly when the lesions are small. It is generally considered that the role of low-cost laboratory tests in the detection of LM is of limited value and of minor importance in comparison with imaging procedures; nevertheless the literature includes controversial results that are sometimes surprisingly good.[3-5]

The aim of the present study was to verify the value of CEA and some biochemical hepatic tests in the pre-operative detection of hepatic metastases in patients with primary colo-rectal cancer.

### MATERIAL AND METHODS

We analyzed retrospectively the records of 278 consecutive patients admitted to the Istituto Nazionale Tumori between January 1982 and December 1983 with primary colo-rectal cancer and who underwent laparotomy.

All cancers were invasive adenocarcinomas. In 38 patients (13.7%) LM were assessed at laparotomy; 6 patients had synchronous extrahepatic metastases and were considered to be in the group of patients without liver metastases. Table 1 reports the main characteristics of the series.

The laboratory tests examined were: plasma CEA, alkaline phosphatase (AP), gamma glutamyl transpeptidase (GGT), lactic dehydrogenase (LDH) and cholinesterase (CHE).

Serum CEA was determined using the radio immunoassay kit supplied by Hoffmann-LaRoche, in which values between 0 and 10 nanograms per milliliter are normal. The methods of determination of liver enzymes and the upper or lower limits of normality used in our laboratory in 1982 and 1983 were the following:

AP: Szasz method; upper limit of normal 170 mU/ml.
GGT: Szasz method; upper limit for male 26 U/1, for female 18 U/1.
LDH: Wroblewski method; upper limit 240 mU/ml.
CHE: Ellman method; lower limit of normal 1900 U/1.

We have considered as pathologic those values exceeding the normal range indicated by these methods. The tests were evaluated by calculating the sensitivity (SE), the specificity (SP), the predictive value of a positive (pathologic) test (PVP), the predictive value of a negative (normal) test (PVN) and the diagnostic accuracy (DA).

SE is defined as the proportion of patients with LM, testing positive (A/A + C), where C is the number of false negative cases. SP is the proportion of patients without LM, testing negative (D/B + D), where B is the number of false positive cases. PVP is the proportion of patients testing positive, found with LM at laparotomy (A/A + B). PVN is the proportion of patients testing negative, proved to be free of LM at laparotomy (D/C + D). DA is defined as the proportion of cases correctly diagnosed by the test (A + D/A + B + C + D), where B + C is the number of cases erroneously diagnosed by the test.

The presence or absence of liver metastases was judged at laparotomy by careful inspection and palpation; biopsy for histology was taken whenever some alteration on the gross examination of the liver was apparent. The extension of liver involvement was classified according to the proposal of Gennari *et al.*[6].

Preoperative liver evaluation was performed during that period at our Institution by means or scintigraphy of ultrasonography.

**Table 1** Main characteristics of the series.

| *Variable* | *No.* | *% M+* | *% M−* |
|---|---|---|---|
| No. of pts | 278 | 14 | 86 |
| Sex Male | 147 | 12 | 88 |
| Female | 131 | 15 | 85 |
| Age Mean | 58.6 | 59.1 | 58.8 |
| Range | 30–86 | 34–86 | 30–82 |
| Site of primary: | | | |
| colon recto-sigmoid | 170 | 12 | 88 |
| rectum | 106 | 15 | 85 |
| multiple | 2 | 50 | 50 |
| Histologic type: | | | |
| well diff. | 40 | 12 | 88 |
| mod. diff. | 204 | 15 | 85 |
| poorly diff. | 11 | 18 | 82 |
| other | 23 | 4 | 96 |
| Dukes A | 30 | 3 | 97 |
| B | 134 | 6 | 94 |
| C | 102 | 24 | 76 |
| undetermined + D (liver excluded) | 12 | 25 | 75 |

### RESULTS

First, we evaluated the relation of each single test to LM and next the impact of all the laboratory tests considered together in each case. Table 2 reports the results of single tests.

The findings of the preoperative liver ultrasonography (US) performed in 113 patients, 22 having LM, have been reported for comparison.

The SE of US was 50%, the SP 90%; the PVP was 55% with a lower number of false positive tests than any laboratory test examined. The PVN was quite similar to the other tests considered and the DA was 82%.

**Table 2** Analysis of single tests.

| *Variable* | *Se.* | *Sp.* | *PVP.* | *PVN.* | *DA* |
|---|---|---|---|---|---|
| CEA | 71% | 70% | 30% | 93% | 70% |
| AP | 32% | 90% | 35% | 89% | 82% |
| GGT | 39% | 85% | 31% | 89% | 78% |
| LDH | 51% | 62% | 18% | 89% | 61% |
| CHE | 44% | 81% | 26% | 90% | 75% |
| US | 50% | 90% | 55% | 88% | 82% |

Se = sensitivity PVP = predictive value of a positive test
Sp = specificity PVN = predictive value of a negative test
DA = diagnostic accuracy

Table 3 analyzes the sensitivity of tests in relation to the metastatic involvement of the liver. The direct connection between the positivity of the tests and the extent of LM is evident. In cases of LM involving less than 25% of the parenchyma, CEA was the most sensitive test (62%, 13 out of 21), whereas US (33%, 4 out of 12), and liver function tests (SE ranging between 10% and 36%) failed frequently to identify or suspect the presence of lesions.

Table 4 reports the range and median values of tests in the presence or absence of metastases. From the observation of median values, we can infer that with the exception of CEA, there was no difference between cases with and without metastases and more than half the cases with LM showed normal values. It is evident that the range of values is different in cases with and without LM, being more extreme in the presence of LM, but we can observe consistent deviations in cases without LM as well, particularly in CEA levels.

In 210 patients, where the evaluation was complete, it was possible to analyze the relationship between all the laboratory tests and the presence or absence of LM (Table 5). When all the tests (CEA and liver function tests) were normal the probability of finding LM proved to be very low: in fact we observed only two false negative cases (PVN 96.7%, with 95% confidence limits of 89%–99%). In these two cases laparotomy revealed H1 metastases: the first had been investigated preoperatively with hepatic CT scan, US and scintigraphy, and the second with scintigraphy, but no examination was able to demonstrate the presence of secondary lesions.

**Table 3** Sensitivity of tests in relation to the extent of liver metastases (Gennari's classification).

| *Variable* | *H1 (<25%) ratio* | *%* | *H2 (25–50%) ratio* | *%* | *H3 (>50%) ratio* | *%* |
|---|---|---|---|---|---|---|
| CEA | 13/21 | 62% | 6/6 | 100% | 6/8 | 75% |
| A.P. | 2/21 | 10% | 2/8 | 25% | 8/9 | 89% |
| GGT | 3/21 | 14% | 3/8 | 38% | 9/9 | 100% |
| LDH | 3/18 | 17% | 6/8 | 75% | 9/9 | 100% |
| CHE | 5/19 | 26% | 4/8 | 50% | 7/9 | 78% |
| U.S. | 4/12 | 33% | 3/5 | 60% | 4/5 | 80% |

**Table 4** Range and median values of tests in the presence or absence of metastases.

| *Variable (standard value)* | *With LM* Range | *With LM* Median value | *Without LM* Range | *Without LM* Median value |
|---|---|---|---|---|
| CEA (<10) | 0–4953 | 41.9 | 0–188 | 2 |
| A.P.(<170) | 16–1552 | 121.5 | 17–352 | 114 |
| GGTm. (<26) | 7–218 | 21 | 4–99 | 21 |
| GGTf. (<18) | 4–1864 | 11.5 | 3–222 | 10 |
| LDH (<240) | 45–1523 | 241 | 55–389 | 223 |
| CHE (>1900) | 1430–3260 | 1990 | 440–4600 | 2430 |

**Table 5** Predictive values of abnormal tests in relation to their number.

| *No. abnormal tests* | *M+/M−* | *PVP* | *PVN* |
|---|---|---|---|
| One | 10/68 | 12.8% | — |
| Two | 6/33 | 15.4% | — |
| Three | 5/17 | 22.7% | — |
| Four | 4/1 | 80% | — |
| Five | 5/0 | 100% | — |
| None | 2/59 | — | * 96.7% |

* 95% confidence limits are 89%, 99%.

When one or more tests were abnormal, a direct relationship between the number of abnormal tests and the detection of LM at laparotomy was evident, but the number of false positive cases was quite high, up to three abnormal tests, and became acceptably low in the presence of 4 or 5 abnormal tests.

With the aim of verifying the accuracy of our intraoperative liver evaluation, the follow-up of patients with all normal tests and no evidence of LM at laparotomy was analyzed. Two patients developed clinical evident LM within the first year of follow-up, one after 10 months and the other after 12 months from operation.

Two other patients had LM in the second year of follow-up, another in the third year and two thereafter.

In all, 7 patients out of 54 (13%) evaluable patients (five were lost to follow-up within the second year) developed LM.

### DISCUSSION

When dealing with new diagnostic methods for the detection of LM, there is often a gap between initial encouraging clinical results and later disappointing results obtained in routine use when retrospective studies are performed. However this gap may stimulate investigation into simple, low-priced and objective tests, to better defining their role and limits. Even with the systematic use of scintigraphy, sonography, CT scan and MNR, the finding of a previously undetected LM at laparotomy, is not an exception in clinical practice, and it is plain that the more the results are unsatisfactory, the more the problem of costs cannot be disregarded. The need to razionalize the use of methods in routine practice is then apparent.

In our retrospective series, 11 out of 22 patients who underwent ultrasonography, (the test used now in routine screening in our Institution), showed LM at laparotomy. The results were even poorer in small metastases (4 out of 12). In other prospective series the sensitivity of ecography is between 70 and 90% [7–10], but more than 50% of our patients with LM only had small lesions.

Moreover, it is well known that the accuracy of routine examination is poorer compared with prospective studies.

The low-cost laboratory tests (CEA and liver function tests) have been widely assessed for their diagnostic accuracy in predicting liver metastases from colo-rectal cancer. It is well known that the CEA test usually increases in the presence of LM and the elevation is related to the extent of metastases [3,11–13]. The main limit of

CEA in the prediction of LM is the high number of false positive cases, since it increases frequently with primary lesions.

As regards liver function tests, the limits are: a — they generally show a low specificity, since they change in a number of other hepatic diseases; b — at the same time they have a low sensitivity, unless the liver is widely involved with metastases.

But the results reported in the literature are rather controversial with regard to this point. Whereas most authors find a good sensitivity for massive involvement of the liver but a number of false negative cases, as high as 90% for small lesions, when testing AP [14–18], GGT [15,16] and LDH [18]. Other investigators find a very high sensitivity (75 to 90%), even for small metastases, when testing AP [3,4] or GGT [4,5].

CEA and liver function tests have been studied in various combinations, sometimes modifying the discriminant values between normal and pathologic. Couples of tests, usually CEA and another, would demonstrate a better accuracy than a single test [3, 11, 19]. In our experience, the accuracy of the surgical evaluation of the liver at laparotomy appeared to be quite good, since no patients showed LM before 10 months from operation and only two showed LM within one year. This is not in conflict with recent reports [20, 22] about the accuracy of ultrasonography in the detection of LM unrecognized by palpation, which is about 17%–29% of cases, if we hypothesize a slow evolution of some small lesions deep in the parenchyma. Therefore, we can suppose that the possible underestimation of LM in this series did not influence the results in a relevant way.

In our series, the CEA test showed a better sensitivity than any other test, ultrasonography included (Table 3). If we considered small lesions (Table 4), the difference in sensitivity was even more apparent. The predictive value of the CEA test positive was, however, poor, whereas if the test was negative the probability of finding LM was low (7%).

We observed that an alteration of liver enzyme values was rather infrequent in the presence of LM (the best results are obtained by LDH with 51%, see also median values in Table 4); consistent with most data in the literature, if LM are small the sensitivity of these tests ranges from 7% to 26%, and improves in direct relation to the extent of the metastases. All the liver function tests show a good prediction when negative, but a poor ability of predicting LM when positive. When we considered the results of all the laboratory tests in each patient (Table 5) we concluded that, whenever all the tests are normal, the probability of finding LM at laparotomy is negligible.

If these tests had been used as a criterion for performing further liver investigations, only 2 patients with LM would have avoided 61 (28.5% of the series) useless examinations. As a matter of fact the 2 cases with LM were undetectable even with imaging procedures.

If one or more of the tests are abnormal, the probability of finding LM at laparotomy is directly related to the number of tests altered; the diagnostic reliability is however very low up to three pathologic tests and becomes satisfactory only with four or five.

It is clear that the role of laboratory tests is exhausted when suspicion is aroused, since the surgeon needs further information about the extent, site, number of metastases and their relationship to biliary and vascular systems, so that an imaging technique is required.

Our results confirm all the limits of laboratory tests in detecting LM but indicate a role for them that is as a first step for hepatic evaluation in a patient with primary colo-rectal cancer. When the tests are normal further liver investigations could probably be avoided.

An imaging procedure could, therefore, be reserved only for the proportion of patients with abnormal tests who must be considered at increased risk of bearing LM, they must then be carefully studied with preoperative tests (US) and eventually with intraoperative US.

### References

1. Hughes K.S., Sugarbaker P.H. (1987) Resection of the liver for metastatic solid tumors in "Surgical treatment of metastatic cancer" Edith Rosenberg S.A., Lippincott J.B. Company. Philadelphia.
2. Chang A.E., Schneider P.D., Sugarbaker P.H., Simpson C., Culnane M. and Steinberg S.M. (1987) A prospective randomized trial of regional versus systemic continuous 5-fluorodeoxyuridine chemotherapy in the treatment of colorectal liver metastases. *Ann. Surg.* **206**, 285–293
3. Tartter P.I., Slater G., Gelerut I. *et al.* (1981) Screening for liver metastases from colorectal cancer with carcinoembryonic antigen and alkaline phosphatase. *Ann. Surg.* **193**, 357–360.
4. Baden H., Andersen B., Angustenberg G. *et al.* (1971) Diagnostic value of GGT and alkaline phosphatase in liver metastases. *Surg. Gynecol. Obstet.* **133**, 769.
5. Aronsen K.F., Nosslin B., Pihl. (1970) The value of gamma-glutamul transpeptidase as a screen test for liver tumour. *Acta. Chir. Scand.* **136**, 17–22.
6. Gennari L., Doci R., Bozzetti F. *et al.* (1982) Proposal for a clinical classification of liver metastases. *Tumori* **68**, 443–449.
7. Snow J.H., Goldstein H.M., Wallace S. (1979) Comparison of scintigraphy, sonography and computed tomography in the evaluation of hepatic imaging in the detection of metastatic neoplasm. *Am. J. Rad.* **132**, 915–918.
8. Smith T.J., Kemeny M.M., Sugarbaker P.H *et al.* (1982) A prospective study of hepatic imaging in the detection of metastatic disease. *Ann. Surg.* **195**, 486–491.
9. Arnaud J.P., Daly R., Leguillon A. *et al.* (1982) Etude comparative de l'ecography et de las scintigraphie dans le depistage des metastases hepatiques. *J. Radiol.* **63**, 97–100.
10. Drum DE. (1978) Optimizing the clinical value of hepatic scintigraphy. *Semin. Nucl. Med.* **8**, 346–357.
11. Kemeny M.M., Sugarbaker P.H., Smith T.J. *et al.* (1982) A prospective analysis of laboratory tests and imaging studies to detect hepatic lesion. *Ann. Surg.* **195**, 163–169.
12. McCartney W.H., Hoffer P.B. (1976) Carcinoembryonic antigen assay in hepatic metastases detection, an adjunct to liver scanning. *JAMA* **236**, 1023–1027.
13. Munjal D., Chawla P.L., Lokich J.J. *et al.* (1976) Carcinoembryonic antigen and phosphohexose isomerase, gamma glutamyl transpeptidase and lactate dehydrogenase levels in patients with and without liver metastases. *Cancer* **37**, 1800–1807.
14. Schaefer J., Schiff L. (1965) Liver function tests in metastatic tumor of the liver: study of 100 cases. *Gastroenterol.* **49**, 360–363.
15. Schreve., Terpostra O.T., Ausema L. *et al.* (1984) Detection of liver metastases. A prospective study comparing liver enzymes, scintigraphy, ultrasonography and computed tomography. *Br. J. Surg.* **71**, 435–438.
16. Graffuer H, Hultberg., Johansson B. *et al.* (1985) Detection of recurrent cancer of the colon-rectum. *J. Surg. Oncol.* **28**, 156–159.
17. Ranson J.H.C., Adams P.K., Localio S.A. (1973) Preoperative assessment for hepatic metastases in carcinoma of the colon and rectum. *Surg. Gynecol. Obstet.* **137**, 435–438.
18. Kemeny M.M., Gantlaume L., Goldberg D.A. (1986) Preoperative staging with computerized axial tomography and biochemical laboratory tests in patients with hepatic metastases. *Ann. Surg.* **203**, 169–172.
19. Steele L., Cooper E.H., Mackay A.M. *et al.* (1974) Combination of CEA and GGT in the study of the evaluation of colorectal cancer. *Br. J. Cancer* **30**, 319–324.
20. Finlay I.G., McArdle C.S. (1986) Occult hepatic metastases in colorectal carcinoma. *Br. J. Surg.* **73**, 732–735.
21. Machi J. Isomoto H., Yamashita Y. *et al.* (1987) Intraoperative ultrasonography in screening for liver metastases from colorectal cancer: comparative accuracy with traditional procedures. *Surgery* **101**, 678–684.

22. Gigot J.F., De Neve De Roden A., Weerts J. *et al.* (1988) Intraoperative ultrasound in the detection of occult metastases in colorectal cancer surgery: a prospective multicentric study. Personal communication 2nd World Congress on Hepato-Pancreato Biliary Surgery, Amsterdam May 29–June 3.

*(Accepted by S. Bengmark on 20 December 1989)*

### INVITED COMMENTARY

"The role of CEA and liver function tests in the detection of hepatic metastases from colo-rectal cancer"

This is a retrospective review of 278 patients admitted to the Institute Nazionale Tumori for primary colorectal cancer over a 2 year period. Thirty eight of these patients were found to have liver metastases on laparotomy and this study analyzes how accurately the preoperative laboratory tests predicted this outcome. Not unexpectedly CEA was the most sensitive at 71%, but not the most specific (70%) since often patients with primary colon cancer will have elevated CEA levels with or without liver metastases. The rest of the liver function tests were much less sensitive ranging from 32–51%. The only imaging test studied was ultrasonography (US) done in 113 of the 278 patients. Even in this more select group US only had a sensitivity of 50%.

When the 38 cases with liver metastases were broken down into the Gennari classification of extension of liver involvement it was clear that the greater the involvement the more sensitive the tests. For instance CEA had 62% sensitivity in the H1 category, versus 100% and 75% in the H2 and H3 categories. For liver function tests the sensitivity plummets when there is minimal disease. Alkaline phosphatase has a 10% sensitivity in the H1 category versus 89% in the H3 group.

The last point brought out by this study dealt with the predictive value of pathologic tests in relation to how many tests were abnormal. Thus in the 5 cases which had all five tests abnormally elevated all five patients had liver metastases thus a predictive value of 100%. If only one test was abnormal the predictive value was 12.8%, if two 15.4%, three 22.7%, and four 80%.

This study very much corroborates a number of previous studies including ones I have published with my colleagues (references in the Bonfanti paper). Although in my prospective work I found a higher sensitivity rate for alkaline phosphatase, CEA and LDH (61%, 83% and 58% respectively) the general conclusions of the studies were the same. CEA was the most sensitive laboratory test. Liver function tests were not sensitive as screening tools especially for minimal liver metastases, but if all or most of the tests were elevated the probability of having liver metastases was much higher. The greater the extent of liver disease the more likely there is some biochemical abnormality.

It seems the ultrasound in the Milan group was unusually unsensitive at 50% even in their more select group while we saw a sensitivity of 61% in a prospective group. I don't know the reason for this difference, although Dr. Bonfanti explains it by the fact that the metastases were very small in his group.

I agree with the conclusion of Dr. Bonfanti *et al.* that if none of the liver function tests or the CEA are elevated further investigation is not warranted. However it must be kept in mind that in my study and the Milan study there were patients with liver metastases and totally normal laboratory tests. The advance of more sensitive imaging procedures will help us in the future to detect disease before operation. A large step in sensitivity has been gained in the combined CT angiogram sometimes called dynamic portography. However this is an invasive test and should be reserved for the situations where all other tests have not been useful. The gold standard still remains exploratory surgery with bimanual palpation of the liver. Intraoperative ultrasound, which Dr. Gennari's group reported on several years ago also increases the surgeons ability to detect metastases at laparotomy especially those small deep lesions which cannot be palpated well and in cases of cirrhosis where palpation is all but useless. Dr. Bonfanti reports that of all the patients with no evidence of liver metastases at laparotomy and normal tests (59 patients) two developed liver metastases at 10 and 12 months after operation, thus reconfirming the accuracy of intraoperative examination. It is disheartening that in 1990 we are still drawing the same conclusions about the same tests as we did in 1982, however we as clinicians have to wait till either monoclonal antibody technology for imaging tests or some more specific biochemical marker comes along so that our preoperative accuracy can rise to the more acceptable range of 85–90 percent. Till then no matter how we try to analyze the data we do not have an acceptable, accurate screening tool for detecting liver metastases in patients with colorectal cancer.

Professor Margaret Kemeny
St Vincent's Hospital
153 West 11th Street
NEW YORK, N.Y. 10014
USA

